# Forensic Assessment of Alcohol Intoxication in Cases of Fatal Road Traffic Accidents in Lithuania

**DOI:** 10.15388/Amed.2024.31.1.22

**Published:** 2024-02-27

**Authors:** Beata Kirstukaitė, Akvilė Paškauskienė, Sigitas Chmieliauskas, Sigitas Laima, Diana Vasiljevaitė, Jurgita Stasiūnienė

**Affiliations:** 1 Faculty of Medicine, Vilnius University, Vilnius, Lithuania; 2 Department of Pathology, Forensic Medicine, Institute of Biomedical Sciences of the Faculty of Medicine of Vilnius University, Vilnius, Lithuania

**Keywords:** deaths, traffic accident, alcohol, autopsy, forensic pathology, mirtis, eismo įvykis, alkoholis, autopsija, teismo medicina

## Abstract

**Background:**

There is strong evidence that alcohol consumption is a significant risk factor for fatal road traffic accidents. It is estimated that the number of alcohol-related road accidents remains high in the past few years in Lithuania. This study aims to examine the prevalence of alcohol in blood samples collected from the autopsy results of road traffic accident victims.

**Materials and methods:**

A retrospective study of 136 road traffic accident victims was performed in State Forensic Medicine Service of Lithuania in the period of 2013 to 2023. We analyzed blood alcohol concentration (BAC) in relation to sex, age, road user type, place and time of the day at death.

**Results:**

31% of the victims were under influence of alcohol at the time of death, with mean BAC 1.99 ± 0.92‰. The mean BAC was 2.16 ± 0.8‰ in male and 1.18 ± 1.12‰ in female group. By the type of road users, 23% of the pedestrians (mean BAC 2.45 ± 0.71‰), 32% of car drivers (mean BAC 2.13 ± 0.75‰), 41% of vehicle passengers (mean BAC of 1.73 ± 1.19‰), 37% of the motorcycle riders (mean BAC of 1.28 ± 0.53‰), 37% of the cyclists (mean BAC of 1.15 ± 0.75‰) were found to be intoxicated during the time of accident. Highest mean blood alcohol concentration was found during the night time hours (9 p. m. – 5 a. m.) 2.28 ± 0.91, comparing to in afternoon hours (12 p. m. – 5 p. m.) 1.49 ± 0.99, evening hours (5 p. m. – 9 p. m.) 2.10 ± 0.73 and morning hours (5 a. m. – 12 p. m.) 1.94 ± 1.00. The mean BAC in road traffic accidents during summer was 1.48 ± 0.71‰, spring 2.25 ± 0.76‰, autumn 2.12 ± 1‰, winter 2.42 ± 1‰.

**Conclusions:**

Alcohol consumption by road users is a significant contributing factor in road traffic accidents and their outcomes in Lithuania.

## Abstract (it)

**Įvadas:**

Labai svarbus veiksnys, dėl kurio dažnai įvyksta mirtinų įvykių, yra neblaivūs kelių eismo dalyviai. Pastaraisiais metais mirčių keliuose, susijusių su alkoholio vartojimu, skaičius Lietuvoje išlieka didelis. Šio tyrimo tikslas – remiantis teismo medicininių tyrimų duomenimis įvertinti žuvusių asmenų alkoholio koncentraciją kraujyje eismo įvykio metu.

**Medžiaga ir metodai:**

Atliktas prospektyvusis tyrimas, į kurį įtraukti 136 žuvę kelių eismo įvykių dalyviai. Tyrimas atliktas Valstybinėje teismo medicinos tarnyboje nuo 2013 iki 2023 metų. Analizuotas etilo alkoholio koncentracijos kraujyje ryšys su aukų lytimi, amžiumi, eismo dalyvio tipu, mirties vieta, paros laiku.

**Rezultatai:**

Tiriamosios imties 31 % žuvusių eismo įvykio metu buvo neblaivūs – etilo alkoholio koncentracijos vidurkis 1,99±0,92 ‰. Vyrų grupės alkoholio koncentracijos kraujyje vidurkis 2,16±0,8 ‰, moterų grupės –1,18±1,12 ‰. Pagal skirtingus eismo dalyvių tipus: 23 % pėsčiųjų (alkoholio koncentracijos kraujyje vidurkis 2,45±0,71 ‰), 32 % lengvųjų automobilių vairuotojų (alkoholio koncentracijos kraujyje vidurkis 2,13±0,75 ‰), 41 % automobilių keleivių (alkoholio koncentracijos kraujyje vidurkis 1,73±1,19 ‰), 37 % motociklo vairuotojų (alkoholio koncentracijos kraujyje vidurkis 1,28±0,53 ‰), 37 % dviratininkų (alkoholio koncentracijos kraujyje vidurkis 1,15±0,75 ‰) buvo neblaivūs eismo įvykio metu. Didžiausias alkoholio koncentracijos kraujyje vidurkis nustatytas nakties laiku (21:00–05:00) 2,28±0,91, palyginti su popietės laiku (12:00–17:00) 1,49±0,99, vakaro laiku (17:00–21:00) 2,10±0,73 ir ryto laiku (05:00– 12:00) 1,94± 1,00. Alkoholio koncentracijos žuvusių vasaros metu kraujyje vidurkis buvo 1,48±0,71 ‰, pavasarį – 2,25±0,76 ‰, rudenį – 2,12±1 ‰, žiemą – 2,42±1 ‰.

**Išvada:**

Dalyvavimas eisme apsvaigus nuo alkoholio yra reikšmingas veiksnys, lemiantis mirtiną eismo įvykių baigtį Lietuvoje.

## Introduction

The World Health Organization reports that road traffic accidents claim 1.35 million lives annually [1], making it the leading cause of death among those aged 5–29. In Europe in 2018, car drivers and passengers accounted for 48% of fatal road traffic accidents, while pedestrians, motorcyclists, and cyclists accounted for 27%, 11%, and 5%, respectively. Alcohol is estimated to be a factor in up to 35% of road accidents [1]. In Lithuania in 2020, drunk drivers caused 240 accidents (11.5% of all accidents caused by drunk drivers) resulting in 20 fatalities (18% of all fatalities). Comparing the statistics for the past fifteen years, there is a clear positive trend: since 2005, the number of accidents caused by drunk drivers has fallen by 72.7%, and the number of fatalities by 79.8% [1]. Driving is a complex task that requires a high level of alertness and the ability to perform multiple tasks simultaneously. Alcohol affects a wide range of cognitive functions necessary for safe participation in road traffic both as a driver and as a pedestrian. Alcohol intoxication reduces vigilance, impairs visual perception, the ability to divide attention, and makes it difficult to react quickly to sudden changes in the environment [2]. According to the Lithuanian Law on Road Safety, the maximum blood alcohol content (BAC) [‰] for standard drivers is 0.4. However, changes in cognition are observed even at low BAC levels of around 0.3‰ [3]. A threshold of 0.5–0.8‰ BAC marks a significant increase in the probability of being involved in a crash [4]. Cognitive deficits are even more pronounced when consuming alcohol rapidly and are associated with more risky driving and greater confidence in driving ability [5]. Compared to a sober driver, the risk of being involved in an accident is significantly higher for a drunk driver, even if the BAC is below the legal limit [2]. There is currently no clear threshold BAC that would cause no impairment to cognitive abilities. However, there is a strong association between BAC and being responsible for a fatal road accident [6]. Studies show that people with a BAC above the legal driving limit have more severe injuries and higher in-hospital mortality after road traffic accidents compared to people who test negative [7]. The main objectives of this study are to investigate the prevalence of alcohol in blood samples collected at autopsy of road traffic accident victims in Lithuania and to provide data for the development of further drink-driving prevention measures.

## Materials and methods

### Study design and data source

The research was conducted as a retrospective study analyzing complete forensic pathology autopsies of road traffic accident victims in the period of 2014 to 2023 completed in The State Forensic Medicine Service of Lithuania. All cases in which circumstances of death included a road traffic accident and the cause of death was injuries sustained in a road traffic accident were included in the study. Full autopsies were performed on all victims.

### Identification of cases

The State Forensic Medicine Service of Lithuania provided autopsy data for 136 cases. Of these, 89 road traffic accident victims died suddenly without receiving any specialized medical treatment, while in the remaining 47 cases the patients were hospitalized, and died in the hospital. Toxicological examinations for alcohol and drugs were performed in each case. In each case, information was provided by the law enforcement agencies, including the possible scene of the crime, the time of death, and presumed mechanism of death.

### Statistical analysis

The collected data were processed using R software. The Shapiro–Wilk test was used to determine whether the data were normally distributed. Student’s t-test was used to assess the statistical significance of differences in continuous variables between the study groups. Differences with *p*-values less than 0.05 were considered statistically significant.

### Toxicology Methods

In all cases, the decedents underwent complete autopsies with tests for ethyl alcohol and alcohol surrogates (methyl, propyl, isopropyl, isobutyl, pentyl, isopentyl alcohols), as well as toxicologic tests to detect drugs and other potent substances in the blood and urine. Headspace gas chromatography was used for alcohol detection.

## Results

The study included 136 victims of road traffic accidents, of which 66.9% (n = 91) were male and 33.1% (n = 45) were female. The mean age and standard deviation of all victims was 48.2 ± 21.4 years. The youngest person was 7 years old, the oldest was 98 years old. The mean age for males was 46.4 ± 19.4 years; for females 51.8 ± 24.8 years. The mean age of fatal accident victims was not statistically significantly different between the sexes (*p* = 0.208). There was no significant difference in mean age of the victims who were influenced by alcohol and sober persons died in road traffic accidents (*p* = 0.137). 31% (n=42) of the fatalities in our study were under influence of alcohol at time of death, with a mean ethyl alcohol concentration of 1.99 ± 0.92‰, a median of 2.27‰, and a maximum concentration of 3.36‰. Male victims of road traffic accidents were more likely to be diagnosed with a more severe BAC (n = 35, i.e. 26%), than female (n = 7). The mean BAC was 2.16 ± 0.8‰ in male and 1.18 ± 1.12‰ in female, with no significantly different mean concentrations, *p* = 0.061 (Table 1).

**Table 1:** General characteristics.

	Total	Male	Female	p value
Victims of road traffic accidents N (%)	136 (100%)	91 (66.9%)	45 (33.1%)	-
Mean age and SD, years	48.2 ± 21.4	46.4 ± 19.4	51.8 ± 24.8	*p* = 0.208
Alcohol intoxicated N (%)	42 (31%)	35 (26%)	7 (5%)	-
Mean BAC and SD (‰)	1.99 ± 0.92 ‰	2.16 ± 0.8 ‰	1.18 ± 1.12 ‰	*p* = 0.061

Abbreviations: BAC – blood alcohol concentration; SD – standard deviation

Most road traffic accident victims died at the scene of accident immediately following accident (n = 86, 6% of the total sample). The mean age of those who died at the scene was 45.5 ± 20.6 years. In this group, as many as 44% of the individuals were under the influence of ethyl alcohol, with a mean BAC of 2.03 ± 0.95‰. 3 people died during transportation from the scene of accident to hospital, mean age of this group was 26.7 ± 17.1 years.

The mean age of the road accident victims who died in hospital (n = 47, 35% of the total sample) was 54.4 ± 21.7 years. In this group, only 6% of the individuals were found to have ethyl alcohol in their blood, with a mean BAC of 1.91 ± 0.35‰.

The circumstances of the deceased in the accident were as follows: n = 56 were pedestrians with a mean age of 55.5 ± 21.7 years. 23% of the pedestrians (n = 13) were found to be intoxicated at the time of death with a mean BAC of 2.45 ± 0.71‰. 48% (n = 27) did not die immediately at the scene of the accident and were admitted to a medical facility where they died on average 178 hours after the accident. n = 34 of the deceased were car drivers with mean age of 42 ± 18.1 years. Of them, 32% (n = 11) were found to be under influence of alcohol at the time of death, with a mean BAC of 2.13 ± 0.75‰. Only n = 6, 18% survived the accident and were admitted to a medical institution where they died on average 128 hours after the accident. n = 27 of the deceased were passengers of an automobile with a mean age of 40.7 ± 23.5 years. 41 % of the passengers (n = 11) were found to be influenced by alcohol at the time of death with a mean BAC of 1.73 ± 1.19‰. 26 % (n = 7) survived the accident and were admitted to a medical institution where they died on average 531 hours after the accident. n = 8 of the deceased were motorcycle riders with a mean age of 39.6 ± 17 years; and n = 3, 37 % of the motorcycle riders were found to be under influence of alcohol at the time of death, with a mean BAC of 1.28 ± 0.53‰. In only one case the victim survived at the scene and was admitted to a medical facility where they died 27 hours after the accident. n = 8 of the road traffic accident fatalities were cyclists, with mean age of 50 ± 19.4 years. 37% (n = 3) of the cyclists were found to be under influence of alcohol at the time of death with a mean BAC of 1.15 ± 0.75‰. 50 % (n = 4) survived at the scene of the accident and were admitted to a medical facility where they died on average 133 hours after the accident. n = 2 of the deceased were tractor drivers with mean age 57.5 ± 2.1 years; one of the two deceased were found to be under influence of alcohol at the time of accident at 2.28‰. All cases were sudden deaths at the scene. In the study sample, one case was a scooter driver aged 50 years and sober, admitted to hospital and survived 175 hours after the accident (Table 2).

**Table 2:** Characteristics by road user type.

	N (%)	Mean age and SD	Alcohol intoxicated (%)	Mean BAC (‰) and SD
Pedestrians	56 (41%)	55.5 ± 21.7	23	2.45 ± 0.71
Car drivers	34 (25%)	43.0 ± 18.1	32	2.13 ± 0.75
Car passengers	27 (20%)	40.7 ± 23.5	41	1.73 ± 1.19
Motorcycle riders	8 (6%)	39.6 ± 17.0	37	1.28 ± 0.53
Cyclists	8 (6%)	50.1 ± 19.4	37	1.15 ± 0,75
Other	3 (2%)	-	-	-

Abbreviations: BAC – blood alcohol concentration; SD – standard deviation

When considering the time of the accident, most of them occurred between 6 a.m. and 10 p.m. (n=113, 83%). Mean BAC in afternoon hours (12 p.m. – 5 p.m.) was 1.49 ± 0.99, evening hours (5 p.m. – 9 p.m.) 2.10 ± 0.73, morning hours (5 a.m. – 12 p.m.) 1.94 ± 1.00, night hours (9 p.m. – 5 a.m.) 2.28 ± 0.91. Comparing these time intervals there were no statistically significant difference in BAC, p > 0.05.

The mean blood ethyl alcohol concentration of those died in a road accident during the summer period was 1.48 ± 0.71‰, spring period 2.25 ± 0.76‰, autumn period 2.12 ± 1‰, winter period 2.42 ± 1‰.

## Discussion

Many previous studies have shown that higher BAC is associated with greater cognitive impairment and a higher risk of being involved in a fatal car accident. Previous studies estimate that 10–60% of road traffic deaths are alcohol-related [8]. According to Statistics Lithuania, 1952 people died in road traffic accidents in Lithuania in the period from 2013 to 2023, of which 702 (36%) deaths were caused by alcohol-impaired road users [9]. In our study, 31% (n=42) of the fatalities were under the influence of alcohol at the time of the death. In a study by Davey et al. 51.1% of drivers who were fatally injured in alcohol-related road traffic accidents had a BAC greater than 0.15‰ [10]. A study in Brazil showed that 36.1% of fatally injured drivers were tested positive for alcohol [11]. In a period from 2004 to 2019 the annual number of alcohol-related traffic deaths in Lithuania was reduced by 85% after stricter alcohol control policies were enacted, limiting the availability of alcohol, increasing taxation, banning advertising of alcohol and more strict drink-driving legislation [12]. The mean BAC of alcohol-related crashes victims in our study was 1.99 ± 0.92‰. The mean concentration is similar to that found in Sweden (1.7‰), but the median in our study was significantly higher (2.28‰ vs. 1.7‰ in Sweden), suggesting that more people were heavily intoxicated [4].

In our study more males were involved in fatal road traffic accidents compared to females (n=91, 66.9% of victims were male). The mean BAC was also found to be significantly higher in males (2.16 ± 0.8‰ vs. 1.18 ± 1.12‰ in females (*p* = 0.061)). This is in line with previous studies showing that male sex is associated with a higher risk of alcohol-related fatal crashes as well as with higher number of fatalities in road traffic incidents in general [4,10,13-15]. In the similar study in Sweden, 83% of fatal road accident victims were male [4]. Higher risk-taking propensity and a higher total alcohol consumption are among the reasons for the higher fatality rate of men in alcohol-related road accidents. A survey conducted in Europe found that men consume 80% of all alcohol consumed [16]. According to a survey conducted by Statistics Lithuania in 2019, Lithuanian men consume alcohol 3.2 times more often than women (20.9% of men consume alcohol every week compared to 6.5% of women) and only 9.9% of men report that they never consume alcohol vs. 11.9% of women [9]. A study by Jiang et al. in Australia found that one liter increase in alcohol consumption *per capita* was associated with an increase in road traffic fatalities of 3.4 for males and 0.5 for females per 100000 people [17]. Females were significantly more likely than males to be fatally injured as passengers in motor vehicles (*p* < 0.001) [14]. In our study, the mean age of males who died in road traffic accidents was younger than females (46.4 vs. 51.8 years), but the difference was not statistically significant (*p* = 0.208).

Previous studies have shown that the presence of alcohol in the blood was most commonly detected in pedestrians with up to 40% of fatalities being alcohol-related [11,14,20]. It is important to note that pedestrians are vulnerable road users. Traffic accidents involving pedestrians may be caused by other road users or may occur because pedestrians are under influence of alcohol and behave carelessly in the traffic. A retrospective analysis of pedestrian traffic victims in the Warsaw area, Poland showed that alcohol was found in more than a half the victims examined, mean BAC 2.05 ± 0.89‰, 67% of them died at the scene [21]. In our study, only 26% of alcohol-impaired pedestrians survived at the accident scene and were taken to the hospital where they died on average after 531 hours. 25% of our sample were car drivers. 32% were found to be intoxicated with a mean BAC of 2.13 ± 0.75‰. Study by Pasnin et al. in Norway showed that 15% of fatally injured car and van drivers had an alcohol concentration above the legal limit at the time of the accident [22]. A study in the United States found that in 2007 one in five car or light truck drivers had a BAC above the legal limit [23]. A study in Norway found that 24% of car and van drivers had BAC above the legal limit [22].

20% of the analyzed road traffic victims were the passengers of light vehicles. 41% of the passengers were found to be under the influence of alcohol at the time of death with a mean BAC of 1.73 ± 1.19‰. We found only a few studies that examined the role of the passengers in fatal car traffic crashes, but the majority of them state that drunk passengers can be considered as a risk factor for a fatal traffic accident to occur as the driver can be socially influenced by passengers [24,25].

In previous reports, 27% to 30% of motorcyclists tested positive for alcohol [18]. Alcohol consumption was also associated with lower helmet use [18,19,26]. Our study included an equal number of cyclists and motorcyclists each accounting for 6% of all victims. Half of them were intoxicated at time of the crash (motorcycle riders’ mean BAC 1.28 ± 0.53‰. vs. cyclists’ mean BAC 1.15 ± 0.75‰). The study by Sethi et al. found that 15.1% of injured cyclists were intoxicated prior to injury. This study also showed that mortality was higher among alcohol-impaired cyclists than in nonimpaired group (2.9% [0.6-8.2] vs. 0.0% [0.0-0.6]) [26].

In our study, blood alcohol was most frequently detected in car passengers (41%), followed by cyclists (37%) and motorcyclists (37%). Researchers in Norway also came to similar conclusions, that BAC was highest in cyclists (43%), pedestrians (24%) and motorcyclists (20%) [22].

Most of the road traffic fatalities included in our study occurred during daytime hours between 6 a.m. and 10 p.m. (n=113, 83%), which may be due to more intense traffic. Meanwhile, a study of European countries by Legrand et al. showed the opposite results – most of the crashes occurred at the nighttime on weekdays or weekends and injured drivers were significantly more likely to be intoxicated [27]. The study in the United States by Fell et al. also found similar results to Legrand et al. that three times more (62%) of fatal car crashes occur at night (6 p.m. – 6 a.m.) than during the day (19%) [23]. Another study conducted in the United States by Romano et al. included only young alcohol-related deaths and found statistically significant results that 15–20 year old passengers were more likely to be riding with alcohol-impaired driver on a weekend night (OR = 8.2, OR = 6.2, respectively) or on a weekday night (OR = 5.2; OR = 3.9, respectively) than on a weekday during the day [25]. In our study, victims who died at night between 10 p.m. and 6 a.m. were more intoxicated than those who died during the day (2.28‰ vs 1.86‰), but the difference was not statistically significant (*p* = 0.175). In previous studies, drivers were more likely to be fatally injured in alcohol-related road traffic accidents during weekends (*p* < 0.001) [11,13].

## Conclusions

Our study shows that alcohol consumption remains a significant contributing factor for deaths in road traffic fatalities in Lithuania. Almost one third of victims were under the influence of alcohol at the time of death. Although significant policy changes have been implemented to reduce road traffic mortality, alcohol consumption remains one of the main causes for fatal accidents in Lithuania.
